# The Real *maccoyii*: Identifying Tuna Sushi with DNA Barcodes – Contrasting Characteristic Attributes and Genetic Distances

**DOI:** 10.1371/journal.pone.0007866

**Published:** 2009-11-18

**Authors:** Jacob H. Lowenstein, George Amato, Sergios-Orestis Kolokotronis

**Affiliations:** 1 Division of Vertebrate Zoology, American Museum of Natural History, New York, New York, United States of America; 2 Department of Ecology, Evolution, and Environmental Biology, Columbia University, New York, New York, United States of America; 3 Sackler Institute for Comparative Genomics, American Museum of Natural History, New York, New York, United States of America; University of Bristol, United Kingdom

## Abstract

**Background:**

The use of DNA barcodes for the identification of described species is one of the least controversial and most promising applications of barcoding. There is no consensus, however, as to what constitutes an appropriate identification standard and most barcoding efforts simply attempt to pair a query sequence with reference sequences and deem identification successful if it falls within the bounds of some pre-established cutoffs using genetic distance. Since the Renaissance, however, most biological classification schemes have relied on the use of diagnostic characters to identify and place species.

**Methodology/Principal Findings:**

Here we developed a cytochrome *c* oxidase subunit I character-based key for the identification of all tuna species of the genus *Thunnus*, and compared its performance with distance-based measures for identification of 68 samples of tuna sushi purchased from 31 restaurants in Manhattan (New York City) and Denver, Colorado. Both the character-based key and GenBank BLAST successfully identified 100% of the tuna samples, while the Barcode of Life Database (BOLD) as well as genetic distance thresholds, and neighbor-joining phylogenetic tree building performed poorly in terms of species identification. A piece of tuna sushi has the potential to be an endangered species, a fraud, or a health hazard. All three of these cases were uncovered in this study. Nineteen restaurant establishments were unable to clarify or misrepresented what species they sold. Five out of nine samples sold as a variant of “white tuna” were not albacore (*T. alalunga*), but escolar (*Lepidocybium flavorunneum*), a gempylid species banned for sale in Italy and Japan due to health concerns. Nineteen samples were northern bluefin tuna (*T. thynnus*) or the critically endangered southern bluefin tuna (*T. maccoyii*), though nine restaurants that sold these species did not state these species on their menus.

**Conclusions/Significance:**

The Convention on International Trade Endangered Species (CITES) requires that listed species must be identifiable in trade. This research fulfills this requirement for tuna, and supports the nomination of northern bluefin tuna for CITES listing in 2010.

*–The question is not what you look at, but what you see*

*Thoreau*

## Introduction

The cognomen “bluefin tuna” encompasses three distinct species: southern bluefin tuna (*Thunnus maccoyii*, Castelnau 1872), Pacific bluefin tuna (*T. orientalis*, Temminck & Schlegel 1844), and northern bluefin tuna (*T. thynnus*, Linnaeus 1758) [1]. As sushi, bluefin are unrivaled in popularity, and the economic value per fish unmatched by any other species [2]. Immediate demand for bluefin has far outpaced efforts for long-term management threatening the persistence of this species triad. As a result, in a recently published sushi advisory guide, a collective of conservation organizations urged consumers to avoid eating bluefin altogether [3]. Efforts to extend the public's appreciation of bluefin beyond sushi highlight iridescent grandeur [4], [5]: fish that can exceed a ton in weight [6], reach speeds of over 50 km/h [7], cross ocean basins [8], depths and temperatures [9]–[11], returning to spawn in the same ancestral waters [12] fished by people for millennia [13]. Efforts to garner reverence for bluefin—and with it a popular prohibition against consuming them—are limited because tuna sushi is often made with less imperiled species. Distinguishing bluefin's smallest essence, its DNA, plays a role in cultivating conscientious consumerism and effective regulation by eliminating market ambiguity.

Molecular forensics provide one of the few means for identifying fish sold in retail both due to insufficient labeling requirements or rampant mislabeling of the product [14]. In the US, the Food and Drug Administration (FDA) maintains a registry of 93 approved market names (http://www.cfsan.fda.gov/~frf/seaintro.html) to protect consumers against economic fraud. Cases of fraud have been best documented in red snapper mislabeling [15]–[18]. Whereas the name “red snapper” is approved for use by the FDA only for the species *Lutjanus campechanus*, all eight members of the genus *Thunnus* are to be sold according to the FDA simply as “tuna.” In total there are only 45 species-specific FDA market names for fish. While molecular identification plays an important role in confirming the identity of these species, it is often the only way of identifying the other 87 species of commercial importance [19].

Several suitable molecular methods for identifying market samples exist [reviewed in 20], [21]. Here we assess the efficacy of using cytochrome *c* oxidase subunit I (*cox1*) for *Thunnus* identification. The Consortium for the Barcode of Life (CBOL) directs a global effort to assemble a *cox1* sequence reference library for every species of fish on earth (http://www.fishbol.org) in an effort to establish this gene as the *de facto* biological identification marker. DNA-based identification was one of the first applications of molecular taxonomy [22], and has been applied towards identifying caviar since the mid-1990s [23]. What differentiates barcoding is the scale of the CBOL effort, and the ultimate vision of making the identification of most life forms on earth, accessible to anyone irrespective of their taxonomic literacy, via a handheld device the size of a cell phone [24]. As the invention of the spectroscope allowed people to identify elemental composition of stars and enriched our understanding of the universe [25], a handheld barcoding device will do so for our understanding of life on Earth.

A study conceived by a pair of high school students documenting mislabeling of sushi [26] augurs the potential for CBOL to enrich our lives, but also highlights several critical issues that must be addressed before barcoding is democratized. The Consortium for the Barcode of Life has developed the Barcode of Life Database (BOLD), an online identification system (IDS) where *cox1* sequences can be entered and identified with the ease of an internet search [27]. One challenge that must be addressed is technological. Obtaining *cox1* sequences to enter into BOLD currently requires the expertise of scientists [18], operating large sequencers costing hundreds of thousands of dollars–a reality that has sometimes been obscured [28].

It is unlikely that sequencing technology can be miniaturized to a handheld device in the near future [29]. A personal barcoder will instead likely use microarrays, utilizing species-specific oligonucleotide probes [30]. If BOLD hopes to facilitate the development of such technology, it will require not only a technical overhaul of the identification methods currently employed by BOLD, but a philosophical shift in how barcodes are interpreted and species identified.

Currently, the Fish-BOL library is roughly one fifth complete and still there are fundamental questions as to how the sequences should be read. Since Cesalpino in the Renaissance [31], most classification schemes have relied on describing species using discrete diagnostic characters [32]. Systematists favor treating nucleotides as any other character [33], [34]. While a character-based paradigm has been in operation since the beginning of DNA-based identification [23], [35], barcoding as commonly practiced (*sensu* Hebert et al. [36]) remains distance-based. In a sense this is phenetics [37] reincarnate, though barcoding is not atheoretical [38] but rather designates identifications based either on similarity thresholds [39] or on phenetic clustering [36] using neighbor-joining methods [40] under the premise that there will be well-defined gaps between intraspecific and interspecific distances [41]. At present the BOLD IDS relies on distance-based identification despite the fact that such metrics have been rejected by the systematics community for over two decades [42]. Distance-based barcoding attempts to identify species as one would use spectroscopy, but unlike elements, taxonomic classifications are hypotheses, not the biotic equivalent to the periodic table of elements, and will often fail because speciation is not linear [43].

Previous barcoding of *Thunnus* reveal the limitations of a distance-based approach. The genus constitutes eight species: blackfin tuna (*T. atlanticus*, Lesson 1831), longtail tuna (*T. tonggol* Bleeker 1851), yellowfin tuna (*T. albacares*, Bonnaterre 1788), bigeye tuna (*T. obesus*, Lowe 1839), albacore (*T. alalunga*, Bonnaterre 1788), and the three aforementioned species of bluefin [1]. Recently speciated taxa with large effective population sizes that are reasonably stable may constitute particular challenges for barcoding [44] and *Thunnus* appear to fulfill these criteria, though the onset of industrialized fishing in the 1950s has eliminated the stability of populations as they became stocks [45]. While the monophyly of the genus is strongly supported [46], phylogenies based on morphology [31], mtDNA [47], and rDNA [48] are not entirely concordant.

Ward and colleagues [49] examined the suitability of *cox1* barcoding for discriminating *Thunnus* species. They found a mean intraspecific Kimura 2-parameter (K2P; [50]) distance of 0.11% and interspecific distance of 1.04%. By constructing a neighbor-joining tree, the authors were able to discriminate all species by the criterion that samples from putative species clustered together. However, only the cluster comprising albacore and Pacific bluefin, tuna had significant bootstrap support. Rubinoff et al. [22] asked “is phylogenetics intrinsic to barcoding or are NJ clusters simply convenient visualizations?” for what could be accomplished by alternative methods such as BLAST scores. This question remains unresolved by phenetic barcoders. Ward et al. were cautious, referring to their results as phenograms rather than phylogenies but nonetheless they compared their results to systematic treatments. They noted Chow et al.'s conclusion that albacore mtDNA has introgressed into the Pacific bluefin tuna genome [47], and 56% support for the subgenus *Neothunnus* proposed by Collette [31], a result that has since been challenged by a more recent publication by Chow et al. [48] based on ribosomal DNA.

Wong and Hanner [18] used BLAST [51] and BOLD to identify market purchased seafood, including four presumed tuna samples, with mixed results. While they discovered the remarkable substitution of the cichlid tilapia (*Oreochromis*) for “white tuna” (presumably albacore), their study failed to assign any of the three tuna samples to the species level. This result is unsurprising because they adopted a 97% similarity threshold, (in reference to work on birds [39] and crustaceans [52]), which is almost thee times the mean interspecific distance of *Thunnus* found by Ward et al. [49]. Even at 100% sequence similarity there was no agreement between databases on the identities of their tuna samples.

Paine et al. [53], [54] developed a hybrid approach to identify scombrids combining nucleotide characters for *cox1* consensus sequences and consensus UPGMA distances with internal transcribed spacer sequences to differentiate closely related species. The use of consensus sequences eliminates the possibility of assessing potential distance thresholds for identification and allows only the use of characters that are shared by all members of a species for identification.

Here we present a molecular sequence character-based key for diagnosing *Thunnus* species, and compare its performance with phenetic approaches for identifying market samples of tuna. A strictly character-based approach has the benefit of being hypothesis-driven, readily compatible with taxonomic classifications, and necessary for the design of microarrays. In addition to serving as another comparison of DNA barcoding methods [22], [41], [44], [55]–[57], this research clarifies which species of tuna are being sold in sushi restaurants, the accuracy of menu listings, and clarifies the identity of samples in instances where menus are vague. The dire state of northern bluefin tuna populations underscores the importance of assessing the luxury trade in tuna.

The current usage of the common name “northern bluefin” obfuscates the sheer abundance of this species in the southern Atlantic less than half a century ago. Following the introduction of industrial long-lining from Japan and purse seining from the US in the late 1950s, catches peaked off Brazil between 1963–1967, and the population collapsed to ecological and economic extinction within a decade [58]. As a result, several nations formed the International Commission for the Conservation of Atlantic Tunas (ICCAT) in 1966 to ensure the long-term viability of remaining stocks [59]. Now stocks in the western Atlantic have collapsed prompting calls for a 5-year moratorium on this fishery [60]. In 2007, the eastern Atlantic catch was 61,000 tons, twice the quota set by ICCAT [61], and in June of 2008 the European Commission closed the fishery two weeks early as the result of France and Italy grossly exceeding their allotted quotas [62], [63]. However, in November 2008, at the conclusion of the biannual commission meeting, ICCAT decided to allow a quota of 22,000 tons in the East Atlantic for 2009 [64]. Contrary to calls to close the fishery completely by its own independent review [65], environmental organizations, industry, and six member nations [66], ICCAT seems determined to perpetuate policies its own review labeled a “travesty of fisheries management.” As this decision is a clear indictment that ICCAT has failed its mandate, Monaco has placed a formal bid to list northern bluefin tuna at the 15^th^ meeting of the Conference of the Parties to CITES (Convention on International Trade in Endangered Species) to be held in March 2010 in Doha, Qatar (http://www.cites.org/eng/news/sundry/2009/CoP15_dates.shtml). For any species to be listed, it must be identifiable in trade [67]. This work hopes to address that prerequisite.

## Materials and Methods

### Reference Sequences

We downloaded 89 *cox1* sequences from GenBank for the eight species of *Thunnus* published in Ward et al. [49] and Paine et al. [53] to serve as reference sequences for use in deriving our character-based key and against which samples could be identified using a neighbor-joining phylogram [40] with Kimura 2-parameter-corrected distances (K2P) [50]. We selected these sequences following the recommendation of an authority on scombrid taxonomy (B. Collette, pers. comm.). We aligned the reference sequences using Geneious 4.5.3 (Biomatters; http://www.geneious.com) and the alignment was trimmed to the 655 bp length of Ward et al.'s [49] sequences.

### Sample Collection

We collected samples between 5 June and 31 December 2008 from sushi restaurants in New York City, New York, and Denver, Colorado. Whenever bluefin or a tuna species was included in a menu, it was purchased. Otherwise, at most places we attempted to purchase both regular tuna (the muscle cuts described in Japanese as *akami*), and fatty tuna (*toro*) when available. When the menu listing was ambiguous as to the species of tuna being sold, the wait staff or chef were asked clarify “what kind of tuna” was being served and if the reply was not a valid name, the question was reiterated as “what species of tuna.” Prior to 14 June 2008, we assumed that all sushi sold as “white tuna” was albacore, so staff were not asked to clarify the species. When the cost was not prohibitive and it was offered, sashimi (a slice of fish with no rice or wasabi) was purchased instead of nigiri sushi to reduce potential contamination due to handling.

### Laboratory Methods

Samples collected from each order were preserved in 95% v/v ethanol and total genomic DNA was extracted using the DNeasy tissue extraction kit (Qiagen) following the manufacturer's protocol. The *cox1* locus was PCR-amplified on a Mastercycler ep Gradient S machine (Eppendorf) in 25 µl reactions using Illustra Ready-To-Go PCR beads (GE Healthcare), 1 µl of DNA extract, and 0.5 µl of each of the following primers: VF2_t1, 5′–TGTAAAACGACGGCCAGTCAACCAACCACAAAGACATTGGCAC–3′, FishF2_t1, 5′–TGTAAAACGACGGCCAGTCGACTAATCATAAAGATATCGGCAC–3′, FishR2_t1, 5′–CAGGAAACAGCTATGACACTTCAGGGTGACCGAAGAATCAGAA–3′, and FR1d_t1, 5′–CAGGAAACAGCTATGACACCTCAGGGTGTCCGAARAAYCARAA–3′ constituting the COI-3 primer cocktail [68]. The following temperature cycling was used: 94°C for 2 min, 35 cycles of 94°C for 30 s, 52°C for 40 s, and 72°C for 1 min, with a final extension at 72°C for 10 min. PCR products were purified using AMPure magnetic beads (Agencourt) and cycle-sequenced using primers M13F (-21), 5′–TGTAAAACGACGGCCAGT–3′, M13R (-27), 5′–CAGGAAACAGCTATGAC–3′ [69], and the BigDye 1.1 Terminator Reaction Mix (Applied Biosystems, Inc.). Sequencing reactions were purified using CleanSEQ (Agencourt) and ran through an ABI 3730xl DNA Analyzer. For tissues that failed to amplify, the above PCR protocol was repeated using the COI-2 cocktail [68]. All *cox1* sequences produced from this study were deposited on NCBI GenBank (see Table 1 for accession nos.) and are also provided as Supplementary Information (Document S1).

**Table 1:** Restaurant data and identification of samples using BLAST, BOLD, and characteristic attributes (CAs).

GenBank accession no.	Sample ID	Highest BLAST pairwise identity	Highest BOLD reference sequence similarity	Highest BOLD all species similarity	Character-based identificationa,b	Consensus identification	Menu listing	Verbal clarifiation	Price/order (US$)
FJ605741	JHL00400	99.8 = SBT^b^	99.84 = SBT	99.84 = SBT	CCCCATATATTGGC→SBT	southern bluefin tuna	bluefin toro		7
FJ605742	JHL00401	99.8 = SBT	99.85 = SBT	99.85 = SBT	CCCCATATATTGGC→SBT	southern bluefin tuna	bluefin toro		7
FJ605743	JHL00402	99.8 = BET	99.69 = BLK	99.8 = BET	CCCCACGCATTGAC→BET	bigeye tuna	chu toro	bigeye	6.5
FJ605744	JHL00403	100 = BET	99.54 = BLK	100 = BET	CCCTACGGATTGAC→BET	bigeye tuna	tuna	yellowfin^d^	3
FJ605745	JHL00404	98.8 = ESC	no match	98.5 = ESC	TTAAACAGACCAGT→No ID	escolar	white tuna	n/a^e^	3
FJ605746	JHL00506	100 = NBT	100 = NBT	100 = NBT	CCTCACATGTTGAC→NBT	northern bluefin tuna	toro	bluefin	5.95 (mp)
FJ605747	JHL00507	100 = BET	99.69 = BLK	100 = BET	CCCCACGCATTGAC→BET	bigeye tuna	tuna	tuna^f^	2.5
FJ605748	JHL00508	99.7 = ESC	no match	99.85 = ESC	CTAAACAGACCAGT→No ID	escolar	white tuna	n/a^e^	2.75
FJ605749	JHL00509	100 = BET	99.69 = BLK	100 = BET	CCCTACGGATTGAC→BET	bigeye tuna	tuna	maguro; tuna	2.5
FJ605750	JHL00510	100 = NBT	100 = NBT	100 = NBT	CCTCACATGTTGAC→NBT	northern bluefin tuna	bluefin toro		8.5
FJ605751	JHL00512	100 = BET	99.69 = BLK	100 = BET	CCCTACGGATTGAC→BET		bigeye tuna	tuna	tuna^f^
FJ605752	JHL00513	100 = NBT	100 = NBT	100 = NBT	CCTCACATGTTGAC→NBT	northern bluefin tuna	toro fatty tuna	bluefin	12
FJ605753	JHL00514	100 = SBT	100 = SBT	100 = SBT	CCCCATATATTAGC→SBT	southern bluefin tuna	fatty tuna	bluefin	5.5
FJ605754	JHL00515	100 = YFT	100 = BLK	100 = YFT, BLK, BET, DOG	CCCCACGTATTGAC→YFT	yellowfin tuna	tuna	tuna^f^	2.75
FJ605755	JHL00516	100 = SBT	100 = SBT	100 = SBT	CCCCATATATTAGC→SBT	southern bluefin tuna	fatty tuna	bluefin	6.5
FJ605756	JHL00517	100 = BET	99.54 = BLK	100 = BET	CCCTACGGATTGAC→BET	bigeye tuna	toro	bluefin	5.95 (mp)
FJ605757	JHL00518	100 = NBT	100 = NBT	100 = NBT	CCTCACATGTTGAC→NBT	northern bluefin tuna	toro: Boston bluefin		15
FJ605758	JHL00519	100 = NBT	100 = NBT	100 = NBT	CCTCACATGTTGAC→NBT	northern bluefin tuna	akami: Boston bluefin		8
FJ605759	JHL00520	100 = NBT	100 = NBT	100 = NBT	CCTCACATGTTGAC→NBT	northern bluefin tuna	fatty tuna sushi	bigeye	10
FJ605760	JHL00521	100 = NBT	100 = NBT	100 = NBT	CCTCACATGTTGAC→NBT	northern bluefin tuna	medium tuna sushi	bigeye	9
FJ605761	JHL00522	99.8 = NBT	98.85 = NBT	98.85 = NBT	CCTCACATGTTGAC→NBT	northern bluefin tuna	tuna sushi	bigeye	4.5
FJ605762	JHL00523	100 = BET	99.69 = BLK	100 = BET	CCCCACGGATTGAC→BET	bigeye tuna	maguro (tuna)	red tuna^c^	5.95
FJ605763	JHL00524	100 = YFT	100 = BLK	100 = YFT, BLK, BET, DOG	CCCCACGTATTGAC→YFT	yellowfin tuna	maguro tuna	bluefin	2.75
FJ605764	JHL00525	100 = NBT	100 = NBT	100 = NBT	CCTCACATGTTGAC→NBT	northern bluefin tuna	fatty tuna (bluefin)		8
FJ605765	JHL00526	100 = BET	99.54 = BLK	100 = BET	CCCTACGGATTGAC→BET	bigeye tuna	medium fatty tuna	bigeye	5
FJ605766	JHL00527	100 = BET	99.54 = BLK	100 = BET	CCCTACGGATTGAC→BET	bigeye tuna	tuna	bigeye	3
FJ605767	JHL00528	100 = ALB	100 = ALB	101 = ALB	CTCCGCATATCAAT→ALB	albacore	seared albacore		3
FJ605768	JHL00529	100 = YFT	100 = BLK	100 = YFT, BLK, BET, DOG	CCCCACGTATTGAC→YFT	yellowfin tuna	tuna	bluefin	4
FJ605769	JHL00530	100 = NBT	100 = NBT	100 = NBT	CCTCACATGTTGAC→NBT	northern bluefin tuna	toro	bluefin	11 (MP)
FJ605770	JHL00531	100 = BET	99.54 = BLK	100 = BET	CCCTACGGATTGAC→BET	bigeye tuna	fatty tuna	bigeye	5
FJ605771	JHL00532	100 = BET	99.54 = BLK	100 = BET	CCCTACGGATTGAC→BET	bigeye tuna	tuna	bigeye	3
FJ605772	JHL00533	100 = BET	99.54 = BLK	100 = BET	CCCTACGGATTGAC→BET	bigeye tuna	tuna	bigeye	7
FJ605773	JHL00534	100 = BET	99.69 = BLK	100 = BET	CCCCACGCATTGAC→BET	bigeye tuna	toro	bigeye	5
FJ605774	JHL00535	100 = BET	99.54 = BLK	100 = BET	CCCTACGGATTGAC→BET	bigeye tuna	tuna	bluefin	3
FJ605775	JHL00536	100 = BET	99.54 = BLK	100 = BET	CCCTACGGATTGAC→BET	bigeye tuna	toro	bluefin	8 (MP)
FJ605776	JHL00537	100 = BET	99.69 = BLK	100 = BET	CCCCACGGATTGAC→BET	bigeye tuna	tuna	bluefin	2.85
FJ605777	JHL00538	100 = SBT	100 = SBT	100 = SBT	CCCCATATATTGGC→SBT	southern bluefin tuna	toro belly tuna	bluefin	5.5
FJ605778	JHL00539	100 = YFT	100 = BLK	100 = YFT, BLK, BET, DOG	CCCCACGTATTGAC→YFT	yellowfin tuna	tuna (maguro)	bluefin	2.5
FJ605779	JHL00540	99.7 = ESC	No Match	99.85 = ESC	TTAAACAGACCAGT→No ID	escolar	white tuna (albacore)		2.25
FJ605780	JHL00541	100 = BET	99.54 = BLK	100 = BET	CCCTACGGATTGAC→BET	bigeye tuna	tuna	bluefin	3
FJ605781	JHL00542	100 = BET	99.54 = BLK	100 = BET	CCCTACGGATTGAC→BET	bigeye tuna	toro	bluefin	5
FJ605782	JHL00543	100 = BET	99.69 = BLK	100 = BET	CCCCACGGATTGAC→BET	bigeye tuna	tuna	bigeye	5.5
FJ605783	JHL00544	100 = BET	99.69 = BLK	100 = BET	CCCCACGGATTGAC→BET	bigeye tuna	tuna	yellowfin	2.9
FJ605784	JHL00545	100 = BET	99.54 = BLK	100 = BET	CCCTACGGATTGAC→BET	bigeye tuna	toro	yellowfin	5.50 (M/P)
FJ605785	JHL00546	99.8 = YFT	99.85 = ALT	99.85 = YFT, BLK, BET, DOG	CCCCACGTATTGAC→YFT	yellowfin tuna	tuna	bigeye	3
FJ605786	JHL00547	100 = BET	99.69 = BLK	100 = BET	CCCCACGGATTGAC→BET	bigeye tuna	tuna	tuna^f^	3
FJ605787	JHL00548	100 = BET	99.69 = BLK	100 = BET	CCCCACGGATTGAC→BET	bigeye tuna	tuna	makerel tuna^g^	2.75
FJ605788	JHL00549	100 = YFT	100 = BLK	100 = YFT, BLK, BET, DOG	CCCCACGTATTGAC→YFT	yellowfin tuna	yellowfin tuna		N/A
FJ605789	JHL00550	100 = BET	99.54 = BLK	100 = BET	CCCTACGGATTGAC→BET	bigeye tuna	tuna (maguro)	red tuna^c^	2.5
FJ605790	JHL00551	100 = BET	99.54 = BLK	100 = BET	CCCTACGGATTGAC→BET	bigeye tuna	tuna (maguro)	red tuna^c^	2.5
FJ605791	JHL00552	100 = YFT	100 = BLK	100 = YFT, BLK, BET, DOG	CCCCACGTATTGAC→YFT	yellowfin tuna	tuna	bigeye	2.9
FJ605792	JHL00553	100 = BET	99.54 = BLK	100 = BET	CCCTACGGATTGAC→BET	bigeye tuna	bigeye toro (M/P)		5
FJ605793	JHL00554	100 = NBT	100 = NBT	100 = NBT	CCTCACATGTTGAC→NBT	northern bluefin tuna	o-toro (M/P)	bluefin	6
FJ605794	JHL00555	99.8 = SBT	99.85 = SBT	99.85 = SBT	CCCCGTATATTGGC→SBT	southern bluefin tuna	bluefin toro		7
FJ605795	JHL00556	100 = SBT	100 = SBT	100 = SBT	CCCCATATATTGGC→ SBT	southern bluefin tuna	fatty tuna/toro	bluefin	12.50 (mp)
FJ605796	JHL00557	99.8 = BET	99.54 = ATL, LON	99.85 = BET	CCCTACGGATTGAC→BET	bigeye tuna	tuna/maguro	bigeye	9.5
FJ605797	JHL00558	100 = ESC	no match	100 = ESC	CTGAACAGACCAGT→No ID	escolar	super white tuna	white tuna, c	8.95
FJ605798	JHL00559	99.7 = ALB	99.69 = ALB	99.69 = ALB	CTCCGCATATCAAT→ALA	albacore	albacore/a-ba-co		8.95
FJ605799	JHL00560	99.8 = PBF	99.85 = PBF	99.85 = PBF	CTCCGCATACCAAT→PBF	Pacific bluefin tuna	baby blue fin		6.50
FJ605800	JHL00561	100 = PBF	100 = PBF	100 = PBF	CTCCGCATACCAAT→ PBF	Pacific bluefin tuna	chu toro	bluefin	9.50
FJ605801	JHL00562	99.8 = PBF	99.85 = PBF	99.85 = PBF	CTCCGCATACCAAT→ PBF	Pacific bluefin tuna	toro	bluefin	13.00
FJ605802	JHL00563	99.8 = BET	99.4 = NBF	99.8 = BET	CCCTACAGATTGAC→ BET	bigeye tuna	maguro (tuna)	bigeye	5.25
FJ605803	JHL00564	100 = BET	99.85 = BLK	100 = BET	CCCCACGGATTGAC→ BET	bigeye tuna	maguro (tuna)	yellowfin	4.50
FJ605804	JHL00565	100 = ALB	100 = ALB	100 = ALB	CTCCGCATATCAAT→ALB	albacore	albacore		4.00
FJ605805	JHL00566	100 = ESC	no match	100 = ESC	TTAAACAGACCAGT→No ID	escolar	white tuna	albacore	5.00
FJ605806	JHL00567	100 = NBF	100 = NBF	100 = NBF	CCTCACATGTTGAC→ NBF	northern bluefin tuna	mid fat tuna	bluefin	6.50
FJ605807	JHL00568	100 = BET	99.69 = BLK	100 = BET	CCCTACGGATTGAC→ BET	bigeye tuna	tuna (maguro)	bigeye	5.25
FJ605808	JHL00569	99.8 = ALB	99.85 = ALB	99.85 = ALB	CTCCGCATATCAAT→ALB	albacore	white tuna (tombo)	albacore	4.50

a Nucleotides 262, 268, 271, 286, 313, 337, 358, 400, 409, 475, 487, 484, 508, and 535 based on the Ward et al. [49] sequences.

b SBT, southern bluefin tuna (*Thunnus maccoyii*); BET, bigeye tuna (*T. obsesus*); BLK, blackfin tuna (*T. atlanticus*); NBT, northern bluefin tuna (*T. thynnus*); ESC, escolar (*Lepidocybium flavobrunneum*); DOG, dogtooth tuna (*Gymnosarda unicolor*); YFT, yellowfin tuna (*T. albacares*); ALB, albacore (*T. alalunga*); PBT, Pacific bluefin tuna (*T. orientalis*).

c This is not a recognized common name.

d An interviewing error using “yellowfin” as a leading question disqualifies this result.

e Prior to 14 June 2008 staff were not asked to clarify the identity of white tuna.

f Though ambiguous, this is the accepted US Food and Drug Administration market name for all members of *Thunnus*.

g This is an uncommon vernacular typically refering to kawakawa (*Euthynnus affinis*; http://www.fishbase.org).

### Sample Identification

We identified the samples using three approaches: characteristic attribute diagnosis, sequence similarity, and K2P distance.

To construct a character-based key we visually inspected the reference sequences for variable nucleotide sites that could serve as diagnostics for the eight species. Sarkar et al. [70] expanding on population aggregation analysis (PAA) [33], named such diagnostics as *characteristic attributes* (CAs) and defined them as “a character state found in one clade but not its sister group.” We adopt Sarkar et al.'s terminology but redefine CAs in our phylogenetic-free context to mean a character state that is unique to a species. A CA can be pure (possessed by all members of a species and absent from all others), or private (possessed by some members of a species but absent from all others). In addition, CAs at a single position are termed “simple CAs,” whereas combinations of characters at multiple positions are termed “compound CAs.” After identifying a sufficient number of CAs to differentiate the eight species represented in our reference sequences, we identified the species origin of our samples by detecting CA sites. We used the P-Elf Perl script [71] to automate the identification of these sequences. For sequence similarity we used NCBI's nucleotide BLAST [51] server against NCBI GenBank (http://blast.ncbi.nlm.nih.gov), and mined the BOLD Identification System [27] (BOLD-IDS; http://www.boldsystems.org) to identify each 655 bp-long sample sequence. The sample was identified as the species with which it shares the highest percent pairwise identity for BLAST, or percent specimen similarity in BOLD. In BOLD we used all species-level barcode records, as well as the reference barcode database, which is deemed validated by the criteria of having at least three sequences of at least 500 bp that show less that 2% divergence [27].

We calculated the pairwise K2P distances using MEGA 4.1 [72] for all 89 reference sequences to find the threshold that would minimize the error of both false positive and false negative identifications [41]. Because the threshold value using all reference sequences is biased due to uneven taxon sampling, we also determined optimal threshold values for five randomly selected individuals of each species. Samples were identified against the 89 reference samples using the two threshold values described as well as the smallest interspecific distance to minimize false negatives. In addition, samples were re-identified by removing 3 reference sequences that were shy of the 500 bp requirement used in the BOLD reference database. An identification was deemed ambiguous if the pairwise distance of the sample was lower than the threshold value for multiple reference species, or if the distance from a query to all reference species was larger than the determined threshold value.

Tree-based identifications were conducted using distance as a phylogenetic optimality criterion and more specifically the neighbor-joining method [40] with the K2P substitution model in MEGA. Node support was evaluated with 1000 bootstrap pseudoreplicates. We deemed an identification successful according to liberal tree-based criteria [73]: a query clusters with conspecifics with a minimum of 50% node support, i.e. a node present on a 50% majority-rule consensus tree. A more conservative approach would identify only those samples that fall within a monophyletic clade [56], [57], [74], and would require higher support values.

## Results

Of the 68 samples from 31 establishments (Table 1), eight were listed on menus as bluefin, 4 as albacore, 1 as bigeye, and 1 as yellowfin tuna. There was no written description as to which species was being served for the other samples. For this latter group, when asked for verbal clarification as to what species was being served, 20 were said to be bluefin (with no indication as to which species), 17 as bigeye tuna, 4 as yellowfin tuna, 1 as dorado (*Coryphaena hippurus*), 3 as “tuna” (ambiguous but correct by the U.S. Food and Drug Administration standards), 3 as “red tuna,” 2 as “white tuna,” and 1 as “mackerel tuna.” One sample (JHL403) was excluded as the interviewer slipped and used a leading question with the example “yellowfin” to query the species. The identity of two pieces identified on the menu as white tuna was not verbally queried. Price per order ranged between US$2.25 and $15 and ranged in mass between 9 and 40 grams.

### Character-Based Identification

**Figure 1:**
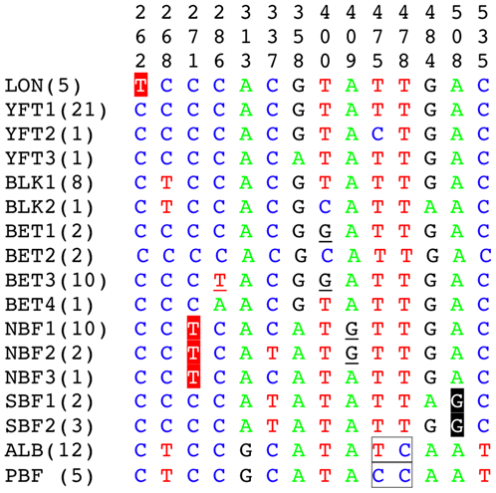
Character-based key for all species of tunas (*Thunnus*) derived from 87 reference sequences [49], [53] constituting 14 nucleotides. Numbers in brackets indicate the number of individual sequences. Nucleotide positions are numbered following Ward et al.'s [49] sequences. Simple pure characteristic attributes (CAs; highlighted) identify longtail (*T. tonggol*; LON), northern bluefin (*T. thynnus*; NBF), and southern bluefin (*T. maccoyii*; SBF) tuna. Simple private CAs (underlined) diagnose bigeye tuna (*T. obesus*; BET). Compound pure CAs diagnose yellowfin tuna (*T. albacares*; YFT), blackfin tuna (*T. atlanticus*; BLK), albacore (*T. alalunga*; ALB), and Pacific bluefin tuna (*T. orientalis*; PBF). Though albacore mtDNA has introgressed into the Pacific bluefin tuna mitogenome, compound pure CAs can differentiate these species (boxed).

We identified 14 diagnostic positions at sites 262, 268, 271, 286, 313, 337, 358, 400, 409, 475, 487, 484, 508, and 535 (Figure 1). The combination of these 14 sites resulted in 17 compound CAs for the eight species. Longtail tuna, albacore, and Pacific bluefin tuna all had a single nucleotide at each diagnostic site, whereas the nucleotides for the other species were not fixed at some positions yielding multiple compound CAs. Longtail tuna, southern bluefin tuna, and northern bluefin tuna could all be identified by a simple pure CA. Compound CAs differentiated the other species.

In constructing the key, we discovered anomalies in 2 reference sequences from Paine et al. [53]. A blackfin tuna sequence (GenBank accession no. DQ835884) had an ambiguous nucleotide (N) at positions 268 and 400 and one bigeye tuna sequence (DQ835863) had CAs identical to blackfin tuna (CTCCACGTATTGAC). We downloaded every publicly available *cox1* bigeye and blackfin tuna sequence from GenBank and BOLD and then using MUSCLE [75] within Geneious ordered sequences by similarity. This sequence (DQ835863) was grouped with blackfin tuna and shared 5 pure simple CAs (Figure S1). These two Paine et al. [53] sequences were not incorporated into the design of our diagnostic key. The character-based key we constructed identified all 63 samples (Table 1). Five samples did not match the CAs for any species of tuna and these samples were subsequently identified by both BLAST and BOLD as escolar (*Lepidocybium flavobrunneum*), a species of gempylid snake mackerel.

### Identification Using BLAST and BOLD

GenBank yielded a definitive top pairwise identity for all samples tested with scores ranging from a low of 99.7% for escolar (*Lepidocybium flavobrunneum*; JHL508 and JHL540), to 100% for all other species (Table 1). Results from the BOLD all species searches were in agreement with the GenBank results, except for samples that BLAST returned as a highest match for yellowfin tuna. Both searches yielded 100% identity, but whereas BLAST returned a single species match, the BOLD all species search yielded a 100% match for four species: bigeye tuna, blackfin tuna, yellowfin tuna, and dogtooth tuna (*Gymnosarda unicolor*), thus yielding ambiguous identifications for 10% of the samples searched. The BOLD reference database matched BLAST and BOLD all species searches for the three bluefin species and albacore. The BOLD reference database failed to return an identification for escolar, and yielded a top match for all samples identified in the other databases as bigeye tuna or yellowfin tuna as blackfin tuna. As a result, the BOLD reference database failed to identify 62% of the samples.

### Identification with Distance Thresholds and Neighbor-Joining Phylogram

**Figure 2:**
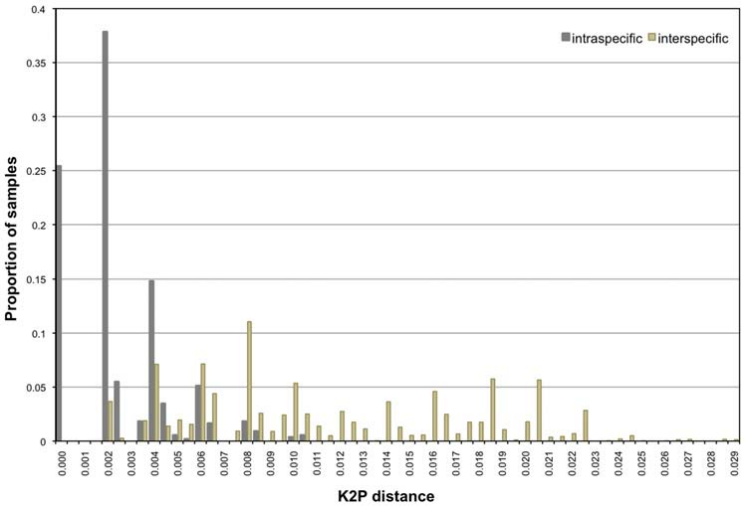
Cumulative intraspecific and interspecific K2P distances for 87 reference sequences. The maximum intraspecific distance was 0.01038 while the minimum interspecific distance was 0.00153.

**Figure 3:**
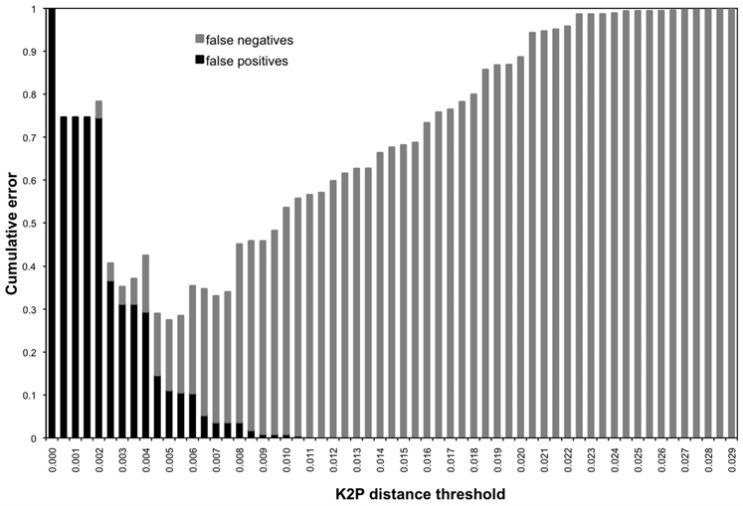
Cumulative error for committing false positives and false negatives with 87 reference samples according to K2P distance thresholds. Error was minimized at 27% at *D*_K2P_ = 0.005.

The mean intraspecific K2P distance for the 87 reference sequences was 0.0022, the mean interspecific distance was 0.012 (Table 2), with no gap between intraspecific and interspecific distances (Figure 2). The two aberrant sequences (DQ835884 and DQ835863) found in constructing the diagnostic key were removed for these calculations. The cumulative error from both false positives and false negatives is minimized at 27% using a threshold value of 0.005 (Figure 3). With even sampling, using five reference sequences for each species, cumulative error is minimized at 31% at *D*_K2P_ = 0.065. The error from false negatives is eliminated at *D*_K2P_>0.0104, and error from false positives is eliminated at *D*_K2P_<0.00153 for both total and even sampling.

**Table 2:** Average K2P distance and diagnostic sites for the eight species comprising the genus *Thunnus*.

Species (sample size)	Albacore	Bigeye tuna	Blackfin tuna	Longtail tuna	Northern bluefin tuna	Pacific bluefin tuna	Southern bluefin tuna	Yellowfin tuna
	Average K2P distance							
**Albacore (12)**	**0.00172**	0.02026	0.01574	0.01877	0.00313	0.01924	0.01773	0.01755
**Bigeye tuna (15)**	268, 286, 313, 358, 400, 478, 484	**0.00313**	0.00807	0.00666	0.01888	0.0106	0.0108	0.00509
**Blackfin tuna (9)**	313, 358, 400, 478, 484, 535	268, 286, 400, 484, 508	**0.00386**	0.00721	0.01616	0.01113	0.01246	0.0052
**Longtail tuna (5)**	262, 268, 313, 358, 478, 484, 535	262, 286, 400	262, 286, 400	**0.00123**	0.01685	0.00947	0.00988	0.00353
**Northern bluefin tuna (13)**	268, 313, 337, 409, 478, 484, 535	271, 286, 337, 358, 400, 409	268, 271, 358, 400, 409, 484	262, 271, 337, 358, 400, 409, 484	**0.00239**	0.01833	0.01494	0.01651
**Pacific bluefin tuna (5)**	475	268, 286, 313, 358, 400, 475, 478, 484, 535	313, 358, 400, 475, 478, 484, 535	262, 268, 313, 358, 475, 478, 484, 535	268, 271, 313, 337, 409, 475, 478, 484, 535	**0.00046**	0.00955	0.00774
**Southern bluefin tuna (5)**	268, 313, 337, 478, 484, 508, 535	286, 337,358, 400, 484, 508	268, 337, 358, 400, 508	262, 337, 358, 484, 508	271, 337, 409, 484, 508	268, 313, 337, 475, 478, 484, 508, 535	**0.00276**	0.00867
**Yellowfin tuna (23)**	268, 313, 358, 475, 478, 484, 535	#	268, 358, 400, 475, 484	262, 358, 475	271, 337, 358, 409, 475	268, 313, 358, 475, 478, 484, 535	337, 358, 475, 484, 508	**0.00195**
	Diagnostic sites							

# Private compound diagnostic. The predominant nucelotide sequence for *T. albacares* is C262 C268 C271 **C286** A313 C337 G358 **T400** A409 T475 T478 G484 A508 C535. The majority of *T. obesus* (n = 10) can be differentiated by **T286 G400**. One *T. obesus* (DQ835865) is differentiated by A286, two are differentiated by **C400** (DQ835861, DQ835862), and two by **G400.**

At *D*_K2P_ = 0.005, 14% of the samples could be identified (excluding those identified by database searches as escolar) while at *D*_K2P_ = 0.0065 no samples were identifiable. Using the threshold that minimized cumulative error at *D*_K2P_ = 0.00152, the optimal threshold, 60 samples (95%) could be identified to the species level, while three samples possessed intraspecific distances from the references greater than this threshold. Without removing the aberrant sequences, 41% of the samples could not be identified with the criteria of an exact match (*D*_K2P_ = 0.0).

**Figure 4:**
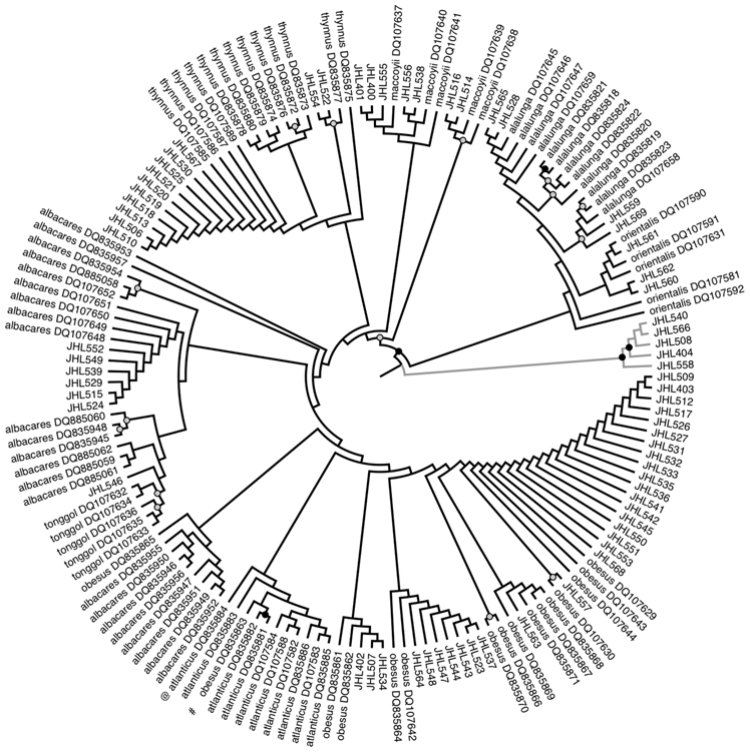
Neighbor-joining tree of *cox1* sequences using the K2P substitution model. Node support was evaluated with 1000 bootstrap pseudoreplicates. Nodes with gray and black circles are supported at 50–79% and 80–100%, respectively. NCBI GenBank accession numbers are included in the taxon label of the reference sequences. The gray clade is composed of escolar sequences.

Our neighbor-joining tree allowed for the identification of 2 samples as albacore, 2 samples as bigeye tuna, 2 samples as northern bluefin tuna, and 2 samples as southern bluefin tuna (12%), but was too poorly resolved to support the identifications of any other samples (Figure 4).

### Identification Consensus

The identifications garnered from our character-based key and the all-species BOLD-IDS were in agreement with our BLAST results, although BOLD was unable to differentiate yellowfin tuna from three other species, and the character-based key made no diagnosis when presented with samples other than tuna. Identification via distance thresholds and neighbor joining were deemed too unreliable for use in identification. Our samples consisted of bigeye tuna (n = 30), northern bluefin tuna (n = 12), yellowfin tuna (n = 7), southern bluefin tuna (n = 7), escolar (n = 5), albacore (n = 4), and Pacific bluefin tuna (n = 3)(Table 1).

Nineteen of 31 restaurants erroneously described or failed to identify the sushi they sold (Table 1). Twenty-two of 68 samples were sold as species that were contradicted by molecular identification (Table 1), while six samples were sold as “tuna” or “red tuna.” While “red tuna” is not considered an approved FDA name, we do not consider this to be a misrepresentation. The five samples of escolar sold as a variant of “white tuna” are considered a misrepresentation because this species is a snake mackerel, belonging to the distantly related family Gempylidae. Except for escolar, no menu listings were contradicted by our analysis. Samples identified as bluefin (0.46 US$/gram) tuna were more expensive (Tukey HSD test, *p*<10^−4^) than either bigeye (0.19 US$/gram) or yellowtail tuna (0.12 US$/gram).

## Discussion

### The Utility of *cox1* for *Thunnus* Identification

This research demonstrates that tuna fish species can be reliably identified with *cox1* barcodes using either our character-based key or highest BLAST sequence similarity. The main limitation of relying on BLAST searches for species identification, however, is the potential for misidentified or low-quality sequences being entered into GenBank [76], [77]. One of the bigeye reference sequences deposited in GenBank that we used (DQ835863) was either mislabeled, misidentified or –at least to our knowledge– the first documented case of introgression between bigeye and blackfin tuna. Although this entry did not affect our BLAST results, there is no way to ensure that BLAST identifications of tuna will remain accurate into the future and should be used only as a first-pass or corroborative identification strategy. Acknowledging this problem, BOLD has a more selective set of submission criteria [27] and has the advantage of using global alignments whereas BLAST uses local alignments. That said, for a variety of reasons outlined below, BOLD performed poorly at identifying tunas.

One of the stated benefits [78] of DNA barcodes is that unlike traditional traits, “they can be obtained in a mechanized manner…used without much background knowledge” [cited in 22]. BOLD is the realization of this philosophy, mechanizing the acquisition of sequences and their analysis. This study, however, illustrates that using the analytical tools supported by BOLD without significant background knowledge may yield results fraught with error even for well-represented taxa such as tuna.

By default, searches on BOLD-IDS use their reference database that is deemed verified according to the following criterion: “species with a minimum of three representatives and a maximum conspecific divergence of two percent” [27]. Searches can also be conducted using all records, or all species records, but these have not been “validated.” According to Ratnasingham and Hebert's description of BOLD [27], it appears that validation requires only that sequences meet the above verification criteria. The BOLD-IDS performs a linear search to collect nearest neighbors of all reference sequences and then delivers an identification if the query sequence shows a match of less than 1% divergence, or in the “few instances” [27] where two or more reference taxa share less than 1% divergence, all possible matches are shown. We retrieved a minimum of 5 candidate species for each of our sushi tuna sample when mining BOLD-IDS. Wong and Hanner [18] identified samples with the BOLD-IDS as the species with which it shared the highest sequence similarity, provided that the distance did not exceed 3%. Using the reference database, they identified presumed yellowfin tuna samples as bigeye tuna, and identified the samples as yellowfin or bigeye using the all-records database. Both databases yielded 100% matches to the queries. Similarly, though their identification criterion was less explicit, Yancy et al. [79] identified two yellowfin samples using the BOLD reference database. During our use of BOLD, (between 10 June 2008 and 4 January 2009), there were no bigeye tuna or yellowfin tuna in the reference database, judged by visualizing the NJ tree output of BOLD-IDS. BOLD-IDS returned blackfin tuna as the highest match on these samples with 99.54–100% similarity to the nearest reference sequence. Because there are multiple bigeye and yellowfin tuna sequences deposited on BOLD it is conceivable that between their analysis and ours someone deposited erroneous sequences for the two species that diverged in excess of 2%, resulting in the removal of the species from the reference database. The lack of transparency in the BOLD system makes this impossible to verify since many of the records that are used for identification are not made public for inspection. Thus, it is impossible to conclude how suspect identifications arose. For example, when we identified yellowfin tuna using the all-species database, BOLD-IDS returned a 100% match for 4 species. While the NJ tree that BOLD-IDS constructs of the top 100 matches could be viewed, none of the sequences for the non-yellowfin nearest-neighbors could be inspected. It was thus impossible to decipher if this result occurred because of problems associated with NJ tree building (discussed below), or because the sequences contained errors that could only be determined by visually examining the nucleotide characters.

A mounting body of work rejects the objectivity and functionality of identification thresholds [22], [55], [57], [74], [80], [81], and our results confirm that the smallest interspecific distance is a more reliable threshold than mean interspecific distance [80]. Using all reference sequences, at *D*_K2P_ = 0.0 we were only able to identify 59% of our samples. Eleven samples exceeded this intraspecific distance, while 15 samples possessed absolute similarity with two species. By conducting a manual pairwise sequence comparison, one reference sequence was responsible for this result. Viewing the blackfin sequence DQ835884.1 revealed that it had been edited with the character “N” at two sites. While this represents an ambiguity of only 0.3% of the entire sequence, these two positions (268 and 400) are critical for distinguishing species (Figure 1). By removing this sequence, we achieved a 95% success rate at *D*_K2P_<0.00153.

Such diagnosis is not possible using BOLD-IDS, and our failure to definitively identify yellowfin tuna using all species records in BOLD likely results from the sequences of the other three candidate species (BOLD accession nos. SAIAB439-06, SCFAC232-06, MXII115-07, SCFAC696-06, SCFAC002-05) being either too short, or poor in quality due to sequencing or editing errors. Thus for BOLD-IDS to work well for tuna, it seems necessary to adopt more selective barcode criteria. This result may also be explained by BOLD's reliance on neighbor-joining to determine the identity of the query to the closest 100 references. Neighbor-joining is an attractive tree-building method because its tree space exploration strategy yields a single best tree, and is computationally fast, but its use has been widely disputed by the systematics and cladistics community [42], [56], [82], [83]. The single tree provided by neighbor-joining is arbitrarily biased due to the order in which sequences are searched in the event of tied-trees [74], [84], [85]. Because the BOLD system does not incorporate any measures of support, its output may be biased and misleading. For instance, the tree constructed using the default parameters in BOLD-IDS for all public *Thunnus* renders yellowfin tuna polyphyletic (Figure S2).

Building consensus trees from bootstrap pseudoreplicates of an alignment can minimize the impact of ties, though in instances where there are many very closely related sequences such as in the case of tuna, ties can still be problematic [86]. Furthermore, bootstrapping combined with neighbor-joining tree-building has been shown to yield artificially high values of support where there are none, a problem that can be avoided by using parsimony jackknifing which is also computationally more efficient [85]. When our queries were paired with our reference sequences, our consensus tree had weaker bootstrap support than the tree presented by Ward et al. [49], due to the addition of more sequences (Figure 3). Even with our liberal identification criteria, we could only identify 12% of the tuna samples using this method, rendering it the worst-performing identification method tested here. Because identification does not hinge on phylogeny [73], and the information content of such phenetic trees is limited and could mislead those without much background information, it seems best to avoid their use.

Contrary to phenetic barcoding, the use of diagnostic characters has at its core the benefit of being visually meaningful, and better approximates a real barcode. Hebert et al. [36] note that just 15 nucleotides yield 4^15^ nucleotide combinations, i.e. barcodes. Distance-based methods reduce the information content of all nucleotides into a single distance vector [34]. For closely related taxa such as tuna, this loss of information renders species diagnosis impossible using the most prevalent identification criteria. The 14 nucleotide sites we selected for our key, however, allow us to differentiate all individuals. Small sequencing errors or ambiguities can potentially have an important impact on identification success under both character-based and phenetic methods. Character diagnosis, however, has the benefit of being hypothesis driven [30], and in that light we were able to reject escolar as a tuna, and to recognize the aberrant sequences downloaded from GenBank.

When tested with additional samples, characters that are critical for species diagnosis may be revealed to be polymorphic and not fixed [33] and it is possible that the key we developed will fail to distinguish species when tested against additional samples. For example, a sample identified as southern bluefin tuna (JHL555; CCCCGTATATTGGC) has a novel polymorphism at position 5, but was still identifiable as it had the species-specific simple pure CA. While this is a limitation of a character-based approach, the fact that these hypotheses can be tested and refined, allows for an objectivity not afforded by distance-based methods. Finally, while there are many concerns about whether barcoding should inform taxonomy [87]–[91], barcodes can be only integrated into taxonomy by using its *lingua franca*: diagnostic characters. A more reciprocal relationship between barcoding and taxonomy could be facilitated if BOLD adopted a character-based approach and systematists made an effort to publish diagnostic barcodes alongside traditional morphological characters [83]. Programs such as P-Gnome automate the discovery of phylogenetically informative CAs [71] and have been used successfully to identify dragonfly [92] and chiton species [93]. A similar program could be incorporated into BOLD to discover diagnostic characters for identification, which could catalyze the design of microarrays [94] and the eventual realization of a handheld barcoder.

### Implications for Consumers and Conservation

The immense profitability of the global demand for sushi and other luxury preparations threatens the long-term persistence of the most coveted species of tuna. In 2001, global imports of chilled or fresh Pacific bluefin, northern bluefin, bigeye and yellowfin tuna was valued at US$935 million [95], while the southern bluefin tuna fishery alone is currently estimated at AU$1 billion (ca. US$754 million at the May 2008 currency conversion rate) [96]. Globally, the stock status for these species ranges from fully exploited to depleted, and the situation will only deteriorate further if catches are not rapidly and significantly reduced [97]. Yet overall demand for sushi is rising [2], despite the deteriorating status of stocks and a growing number of health concerns regarding foodborne toxins and parasites[98]–[101]. Molecular identification serves as an important tool for conservation [67], consumer advocacy [102], and human health [103].

DNA barcodes could serve as a valuable routine tool for use by regulatory agencies concerned with investigating cases of food-related illness or economic fraud [79]. Our study documents five cases of escolar being sold under the name “white tuna”, “white tuna (albacore),” and “super white tuna.” Escolar is banned for sale in Japan and Italy because it contains high levels of wax esters that can cause considerable gastrointestinal distress [104]. According to Shadbolt et al. [105] “symptoms range from mild and rapid passage of oily yellow or orange droplets, to severe diarrhea with nausea and vomiting. The milder symptoms have been referred to as *keriorrhea* [i.e. *flow of wax* in Greek]”. While it is not illegal to sell escolar in the US, and this is not an unambiguous case of economic fraud, the potential consequences of this mislabeling are clearly troubling.

Because all tuna species may be sold under the FDA market name “tuna,” it is also legally tenuous to define substitutions as fraud under existing U.S. regulations. While the majority of bluefin end up as sushi or sashimi in the luxury market, they compose only about 1% of the volume of the principal market species of tuna caught [106]. Bigeye and yellowfin tuna, and to a lesser extent albacore, are also widely consumed as sushi [95]. Of these, albacore that has a whiter flesh is the least substitutable [107]. The fat content of the species typically sold as maguro or “tuna” sushi influences their desirability: bluefin can have up to 15% w/w fat content, bigeye 8%, and yellowfin tuna 2%, though the fat content of species can overlap [108]. The price-to-mass ratio for bluefin, bigeye, and yellowfin tuna based on 2007 US import statistics is 2.4∶1∶1 [13]. In our samples, those that were identified as bluefin using DNA barcodes were significantly more expensive than reflecting the disparity in import value. This all suggests that the FDA should adopt the market name of bluefin tuna to protect consumers against economic fraud. In our survey, four-fifths (79%) of the menu listings gave no indication of what species was being served. When the chef or wait-staff were asked, 32% of the species descriptions were wrong, while 9% of the descriptions were uninformative. Nine of the 22 samples described by restaurants as bluefin, were identified as a different species. Because the generic description of tuna is what customers are accustomed to, it is unlikely that the majority of the failed descriptions were motivated by outright deceit, particularly in instances when the wait-staff –not chef– were relied upon for clarification. From a consumer standpoint it is perhaps reassuring that all 8 of the samples listed in menus as bluefin are identified as such. Fourteen samples from our survey, however, were identified as bluefin tuna without being indicated as such on the menus, thus giving cause for concern given their imperiled status. The only way for consumers to positively avoid consuming bluefin is abstinence from tuna sushi if the verbal confirmations we found is representative.

At a single upscale restaurant we documented the substitution of bluefin for an order of “fatty tuna,” “medium tuna,” and “tuna,” all of which were authoritatively confirmed by the maître d'hôtel as bigeye tuna. This economically counterintuitive result may have occurred because during the time of purchase an exposé by Greenpeace using DNA identification revealed that the Michelin-starred sushi chain Nobu was serving bluefin at three of its London restaurants without informing its customers, and this prompted considerable press coverage and public uproar. As a result, Nobu now lists bluefin on the menus [109] at two of its London restaurants (out of 19 franchises worldwide). Nobu (NYC) was included in our survey and the pieces they sold as bigeye tuna were confirmed as such by our analysis.

At the 2008 ICCAT meeting, the World Wildlife Foundation presented a petition to officials with nearly16000 consumer signatures calling for the boycott of Mediterranean bluefin [110]. While the gesture did not sway ICATT, a widespread boycott of establishments that do not accurately designate the species they serve could prod restaurants to follow Nobu's lead. A boycott successfully catalyzed industry reform with dolphin-safe tuna [111], and perhaps could also cultivate a movement towards bluefin-safe tuna [112].

Both northern and southern bluefin tuna require a level of regulatory urgency unlikely to be met by a consumer movement, necessitating trade restrictions. The history of the two fisheries [59] mirror each other to such an extent that it seems improbable that status quo management for both species will reverse their continued decline. Like the western population of northern bluefin tuna, southern bluefin tuna catches peaked in the 1960s and then collapsed to their present state, whereas 2008 spawning stock biomass was only 10% of pre-exploitation estimates [113]. As with the formation of ICCAT, the collapse spurred concerned nations to form the Commission for the Conservation of southern bluefin tuna (CCSBT) and by 1996 the southern bluefin tuna was declared critically endangered [114]. Like ICCAT, CCSBT admits that, given “estimates of depletion of the spawning stock biomass…the CCSBT has not been successful in managing SBT” [115] but continues to ignore the recommendations of its own scientists, and does not adequately enforce quotas that have been flouted egregiously [116]. As shortsighted economic interests derailed a motion to list northern bluefin tuna on CITES in 1991 [117], the same occurred for southern bluefin tuna in 1999 [118]. Unlike southern bluefin tuna, however, northern bluefin tuna populations have yet to collapse globally.

Our research demonstrates that the technical requirements for CITES listing can be met, and we support the nomination of northern bluefin tuna to the Annex I list of threatened species in order to obviate a fate that seems destined to repeat that of the southern bluefin tuna.

## Supporting Information

Document S1 FastA file with all *cox1* sequences produced from this study.
(0.04 MB TXT)

Figure S1 Alignment comprising all publicly available sequence records available for blackfin (*Thunnus atlanticus*) and bigeye tuna (*T. obesus*) in GenBank and the Barcode of Life Database. Sequence DQ835863.1 appears to be either a case of introgression or a data accession error.
(4.19 MB PNG)

Figure S2 Neighbor-joining tree using the K2P substitution model built in BOLD-IDS using all publicly available *Thunnus cox1* sequences. Note that yellowfin tuna (*T. albacares*) is polyphyletic.
(0.02 MB PDF)
