# Supplementary figures and images for: The Real maccoyii: Identifying Tuna Sushi with DNA Barcodes – Contrasting Characteristic Attributes and Genetic Distances
Source: PLoS One. 2009 Nov 18;4(11):e7866. doi: 10.1371/journal.pone.0007866 (PMC2773415; doi:10.1371/journal.pone.0007866)

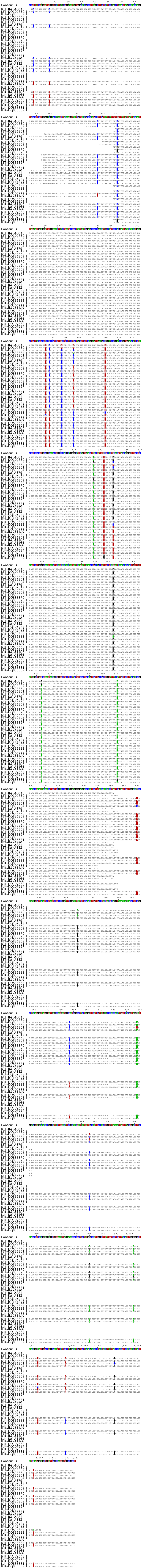

Supplement: Figure S1 — Alignment comprising all publicly available sequence records available for blackfin (Thunnus atlanticus) and bigeye tuna (T. obesus) in GenBank and the Barcode of Life Database. Sequence DQ835863.1 appears to be either a case of introgression or a data accession error. (4.19 MB PNG) [file pone.0007866.s002.png]
