# Supplementary material for: The Real maccoyii: Identifying Tuna Sushi with DNA Barcodes – Contrasting Characteristic Attributes and Genetic Distances
Source: PLoS One. 2009 Nov 18;4(11):e7866. doi: 10.1371/journal.pone.0007866 (PMC2773415; doi:10.1371/journal.pone.0007866)
Supplement: Figure S2 — Neighbor-joining tree using the K2P substitution model built in BOLD-IDS using all publicly available Thunnus cox1 sequences. Note that yellowfin tuna (T. albacares) is polyphyletic. (0.02 MB PDF) [file pone.0007866.s003.pdf]

BOLD TaxonID Tree

Project	:	BOLD Search[NO CODE]
Date	:	26-December-2008
Data Type	:	Nucleotide
Distance Model	:	Kimura 2 Parameter
Codon Positions	:	1st, 2nd, 3rd
Labels	:	SampleID, Sequence Length,
Colorization	:	

Sequence Count	:	51
Species count	:	8
Genus count	:	1
Family count	:	1
Unidentified	:	0

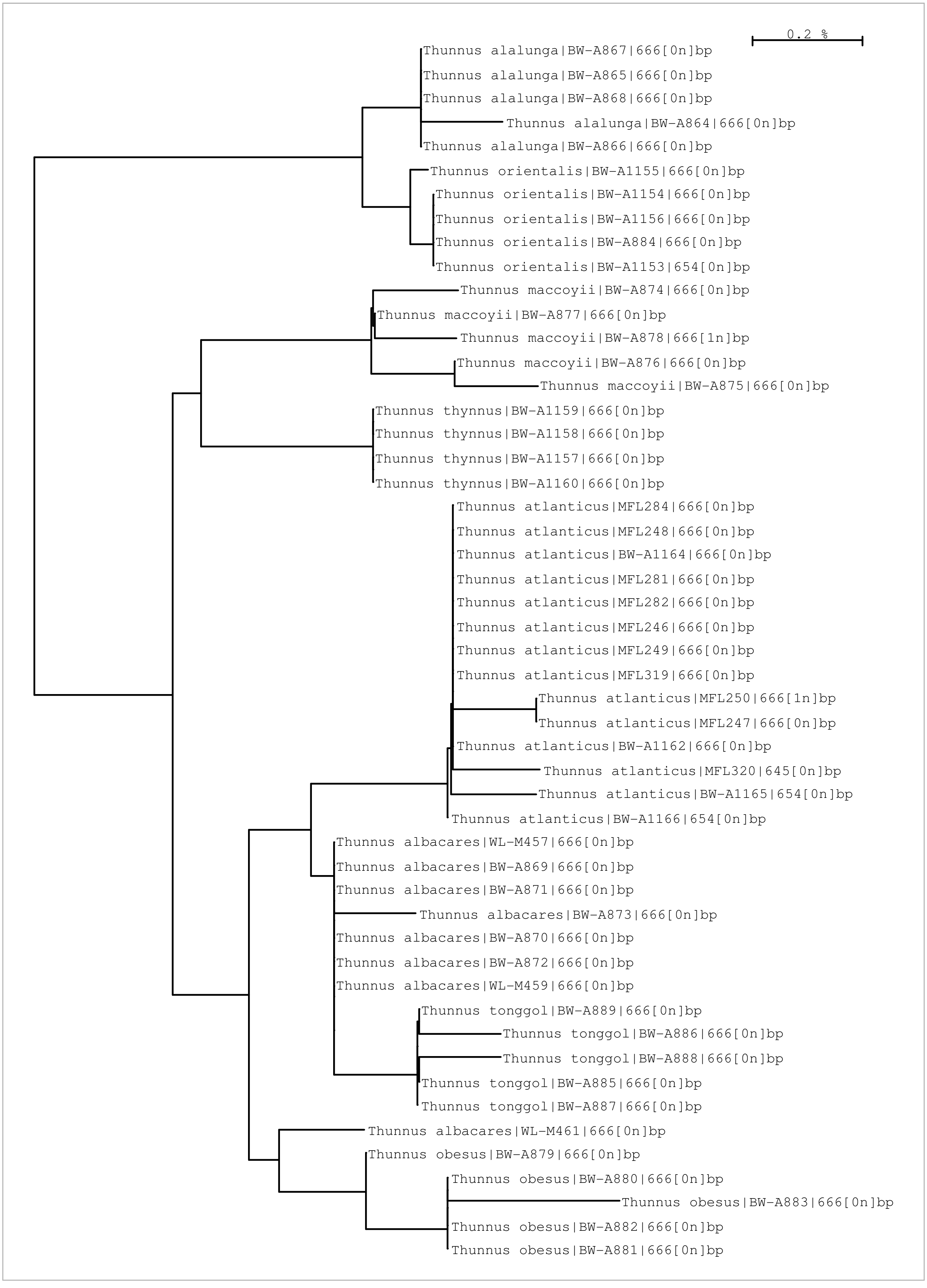
0.2 %
Thunnus alalunga|BW-A867|666[0n]bp
Thunnus alalunga|BW-A865|666[0n]bp
Thunnus alalunga|BW-A868|666[0n]bp
Thunnus alalunga|BW-A864|666[0n]bp
Thunnus alalunga|BW-A866|666[0n]bp
Thunnus orientalis|BW-A1155|666[0n]bp
Thunnus orientalis|BW-A1154|666[0n]bp
Thunnus orientalis|BW-A1156|666[0n]bp
Thunnus orientalis|BW-A884|666[0n]bp
Thunnus orientalis|BW-A1153|654[0n]bp
Thunnus maccoyii|BW-A874|666[0n]bp
Thunnus maccoyii|BW-A877|666[0n]bp
Thunnus maccoyii|BW-A878|666[1n]bp
Thunnus maccoyii|BW-A876|666[0n]bp
Thunnus maccoyii|BW-A875|666[0n]bp
Thunnus thynnus|BW-A1159|666[0n]bp
Thunnus thynnus|BW-A1158|666[0n]bp
Thunnus thynnus|BW-A1157|666[0n]bp
Thunnus thynnus|BW-A1160|666[0n]bp
Thunnus atlanticus|MFL284|666[0n]bp
Thunnus atlanticus|MFL248|666[0n]bp
Thunnus atlanticus|BW-A1164|666[0n]bp
Thunnus atlanticus|MFL281|666[0n]bp
Thunnus atlanticus|MFL282|666[0n]bp
Thunnus atlanticus|MFL246|666[0n]bp
Thunnus atlanticus|MFL249|666[0n]bp
Thunnus atlanticus|MFL319|666[0n]bp
Thunnus atlanticus|MFL250|666[1n]bp
Thunnus atlanticus|MFL247|666[0n]bp
Thunnus atlanticus|BW-A1162|666[0n]bp
Thunnus atlanticus|MFL320|645[0n]bp
Thunnus atlanticus|BW-A1165|654[0n]bp
Thunnus atlanticus|BW-A1166|654[0n]bp
Thunnus albacares|WL-M457|666[0n]bp
Thunnus albacares|BW-A869|666[0n]bp
Thunnus albacares|BW-A871|666[0n]bp
Thunnus albacares|BW-A873|666[0n]bp
Thunnus albacares|BW-A870|666[0n]bp
Thunnus albacares|BW-A872|666[0n]bp
Thunnus albacares|WL-M459|666[0n]bp
Thunnus tonggol|BW-A889|666[0n]bp
Thunnus tonggol|BW-A886|666[0n]bp
Thunnus tonggol|BW-A888|666[0n]bp
Thunnus tonggol|BW-A885|666[0n]bp
Thunnus tonggol|BW-A887|666[0n]bp
Thunnus albacares|WL-M461|666[0n]bp
Thunnus obesus|BW-A879|666[0n]bp
Thunnus obesus|BW-A880|666[0n]bp
Thunnus obesus|BW-A883|666[0n]bp
Thunnus obesus|BW-A882|666[0n]bp
Thunnus obesus|BW-A881|666[0n]bp
